# Pneumorachis mimicking lumbar disc herniation combined with lumbar spinal stenosis: A case report and literature review

**DOI:** 10.1097/MD.0000000000041012

**Published:** 2024-12-20

**Authors:** Songning Fu, Lu Liu, Yadong Liu, Feng Xu

**Affiliations:** a Department of Spine Surgery, The First Hospital of Jilin University; b Department of Stomatology, China-Japan Union Hospital of Jilin University, Changchun, JIlin, China.

**Keywords:** intervertebral disc displacement, misdiagnosis, pneumorrhachis, spinal stenosis

## Abstract

**Rationale:**

Pneumorachis is an uncommon lesion of the spinal canal, which is often asymptomatic. The pathogenesis and treatment strategies are uncertain because only a few cases have been reported. Some patients were treated with percutaneous aspiration or percutaneous endoscopic treatment, but poor pain release and symptom recurrence were observed. Some patients were treated with open surgery and completely cystectomy and had good clinical outcomes. This article reports a case of pneumorachis resembling lumbar disc herniation combined with lumbar spinal stenosis, in which the patient’s compressive symptoms were completely alleviated through open surgery.

**Patient concerns:**

A-56-year-old patient has a 1-year history of radicular pain in the left leg. Magnetic resonance imaging prior to surgery showed a low-signal mass, like a sequestrated disc, in the epidural space at the level of L4 left recess and lumbar spinal stenosis of L4-5 level on both sides. There was a disc-like lesion located at the L4 recess. Computed tomography (CT) showed a homogeneous pneumorachis with a clear boundary. The patient underwent open surgery. Postoperative CT showed that the lumbar canal was decompressed, and this patient was free from pain.

**Diagnoses:**

L4-5 lumbar canal stenosis, pneumorrhachis.

**Interventions:**

For treatment, the patient underwent open surgery to decompress the lumbar canal stenosis.

**Outcomes:**

Postoperative CT demonstrated complete decompression of the L4-5 spinal canal, resulting in immediate relief of the patient’s nerve root pain. At the 3-month follow-up after surgery, the patient remained pain-free.

**Lessons:**

For patients with suspected spinal pneumatosis, CT scans hold significant value to distinguish air and bones from soft-tissue lesions. Furthermore, in this case, we have demonstrated the effectiveness of open surgical treatment for spinal pneumatosis and achieved favorable prognostic outcomes.

## 1. Introduction

Lower extremities radicular pain is often caused by nerve root compression. The common cause is lumbar disc herniation (LDH). Sometimes, gas may accumulate in epidural space (i.e., pneumorachis) and mimics the signs and symptoms of LDH.^[1]^ Pneumorachis is rare, and only limited cases have been reported. Most cases are asymptomatic.^[2]^ The pathogenesis and treatment strategies are uncertain because only a few cases have been reported.^[1,3]^ Some patients were treated with percutaneous aspiration or percutaneous endoscopic treatment, but poor pain release and symptom recurrence were observed; some patients were treated with open surgery and complete cystectomy, and had good clinical outcomes.^[1,3]^

In magnetic resonance imaging (MRI) examination, pneumorachis is similar to LDH, and misdiagnosis is common.^[3]^ Nevertheless, pneumorachis may occur at the same intervertebral level as an actual LDH or lumbar stenosis, leading to misdiagnosis and even maybe inappropriate surgical decision making. Here, we report 1 patient with severe radicular pain caused by lumbar nerve root canal stenosis and pneumorachis at the same level, and treated with open surgery.

## 2. Case report

A 56-year-old Chinese Han man presented at the outpatient department of orthopedics of the First Hospital of Jilin University on December 10, 2018, with a low back pain history of 7 years and a 1-year history of severe left radicular pain to the ankle and numbness of left lateral crural region. Walking would exacerbate the radicular pain and numbness, while bed rest or oral painkillers may alleviate the symptoms. The patient underwent “block therapy” when the radicular pain occurred the first time; the radicular pain was relieved, but the patient could not recall the details. The patient consulted again because the pain was getting worse again. The straight leg raising test was positive at 45° on the left side and negative on the right side. Left long toe extensor muscle strength was decreased, and numbness at lateral crural region was noted. The rest muscle strength and sensation were normal.

**Figure 1.**
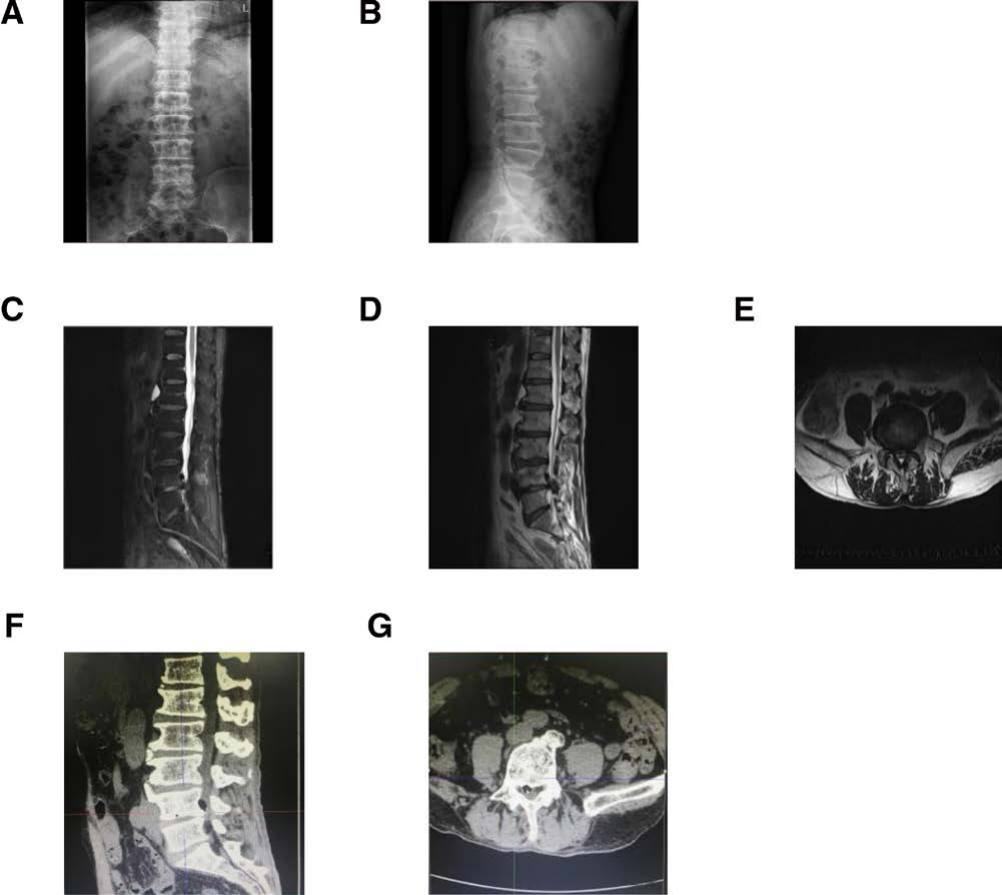
Preoperative X-ray, CT and MRI. (A) Anterior-posterior X-ray view; (B) lateral X-ray view; (C) T2WI image of MRI in median sagittal section; (D) T2WI image of MRI in median sagittal section; (E) T2WI image of MRI in transverse section; (F) CT scan in median sagittal section; (G) CT scan in transverse section. Before admission, the patient underwent MRI. The T2WI images showed a low-signal mass, like a sequestrated disc, in the epidural space at the level of L4 left recess, and lumbar spinal stenosis of L4-5 level on both sides. There was a disc-like lesion located at the L4 recess. After admission, CT showed a homogeneous pneumorachis with a clear boundary, and the density was −931 HU. No vacuum phenomenon was seen in the lumbar disc. CT = computed tomography, MRI = magnetic resonance imaging.

Before admission, the patient underwent MRI. The T2WI images showed a low-signal mass, like a sequestrated disc, in the epidural space at the level of L4 left recess, and lumbar spinal stenosis of L4-5 level on both sides. There was a disc-like lesion located at the L4 recess (Fig. 1).

After admission, computed tomography (CT) showed a homogeneous pneumorachis with a clear boundary, and the density was −931 HU. No vacuum phenomenon was seen in the lumbar disc (Fig. 1).

The patient’s symptoms implied left L5 nerve root compression, but the pneumorachis was observed at the right side, and the L5 nerve root canal stenosis was bilateral. The imaging could not explain the symptoms of the patient. MRI showed that the L4-5 lumbar canal stenosis was narrower on the right side than on the left side. The selective nerve root block was performed on December 13, 2018, to identify the location of nerve compression. We first blocked the left L4-5 intervertebral foramen with 0.5 mL of 1% lidocaine under C-arm X-ray guidance, and the patient’s radicular pain was completely relieved. Hence, after the nerve root block, we were sure of the location of nerve root compression, which was at the left L5 nerve root, even though the MRI showed that the right nerve canal narrower. Therefore, the pneumorachis, in this case, was probably asymptomatic.

We performed open surgery to decompress the lumbar canal stenosis and to determine the nature of the gas-like lesion on December 14, 2018. The patient was placed in a prone position. After lamina exposure, we removed the spinous process, lamina, left L4 inferior articular process, and apex of L5 superior articular process. We found that the lumbar stenosis was severe at the left lumbar canal, but at the right side of the lumbar canal, we found nothing causing compression. We did not find any membranous structure or cyst that could explain why the asymptomatic gas-like lesion. It was hypothesized that the pneumorachis was possibly caused by the first unclear nerve block therapy.

**Figure 2.**
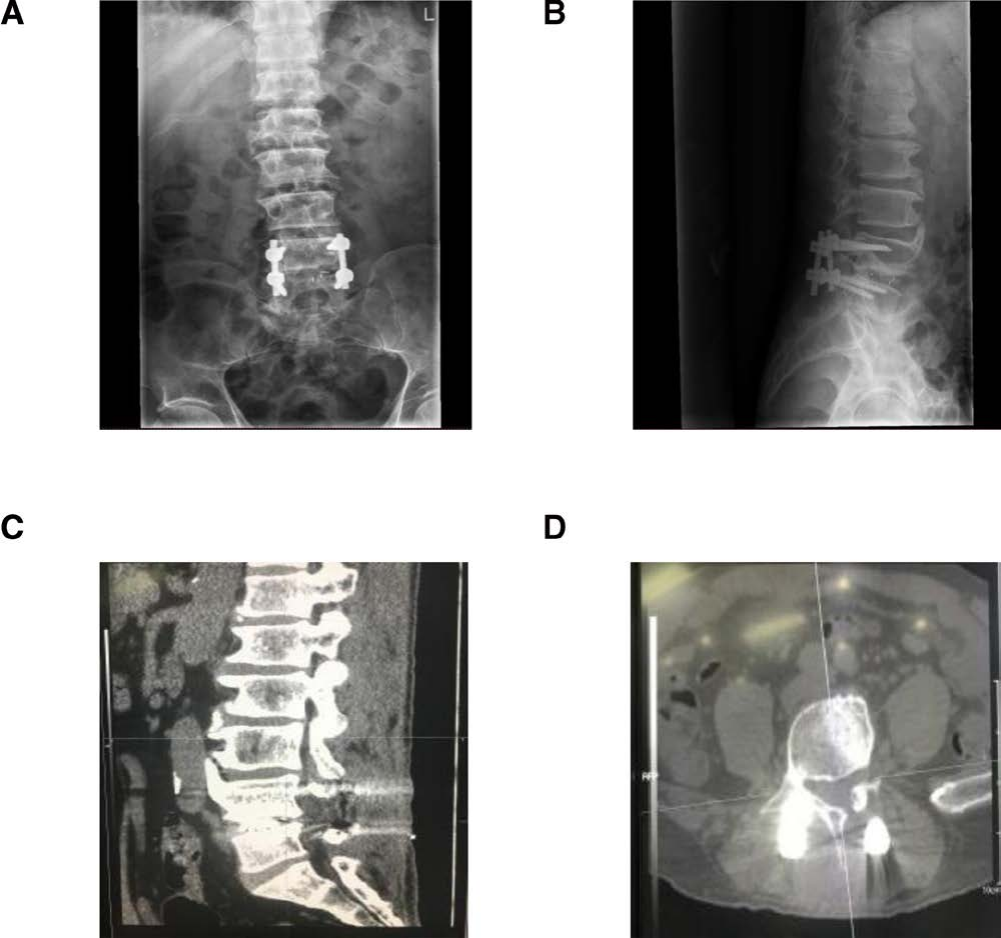
Postoperative X-ray and computed tomography scan. (A) Anterior-posterior X-ray view; (B) lateral X-ray view; (C) median sagittal section; (D) transverse section. Postoperative computed tomography scan showed complete decompression of the L4-5 level of the spinal canal.

The radicular pain was immediately alleviated after surgery. The postoperative CT scan showed complete decompression of the L4-5 level of the spinal canal (Fig. 2). No pneumorachis epidural lesion could be observed in the spinal canal. The patient remained pain-free at the last follow-up at 3 months after surgery.

## 3. Discussion

Pneumorachis is an uncommon lesion of the spinal canal, which is often asymptomatic.^[4]^ We report a patient who had a pneumorachis and lumbar stenosis at the same level. The patient underwent open surgery. Postoperative CT showed that the lumbar canal was decompressed, and this patient was free from pain.

Pneumorachis encompasses any gas lesion in the spinal canal.^[1]^ The exact pathogenesis is unknown, but 3 causes are possible: air in the degenerative disc, and it migrates in the canal following movement and disc prolapse; previous invasive procedure^[5–11]^; or gas produced by an infection.^[1,3]^ Most cases of pneumorachis are asymptomatic.^[2]^ Most reported cases are pneumorachis co-existing with sequestrated disc fragments or capsuled by a cyst.^[1–3,12,13]^ Without a CT scan, it is hard to distinguish epidural gas at the vertebrae recess from free disc based on MRI examination. In the case presented here, it was hard to make the correct diagnosis since epidural gas and lumbar stenosis occurred at the same level. MRI could not identify the gas correctly. Further examinations were needed to make the most appropriate surgical plan, like CT and selective nerve root block or intervertebral foramen block, as in the present study.^[14,15]^

To achieve full decompression of the lumbar stenosis, several options are available, like open surgery and percutaneous endoscopic lumbar discectomy (PELD). PELD is a minimally invasive surgery that now allows solving more severe lumbar spinal stenosis than before, but PELD can sometimes be a dangerous procedure that may cause nerve root or dura damage or insufficient decompression.^[16]^ In addition, in the present case, we sought to determine the nature of the gas lesion. Furthermore, the gas lesion was contralateral to the culprit nerve root, and PELD is a unilateral procedure. Lee et al^[17]^ reviewed 8 cases of symptomatic postoperative pneumorachis from the literature and found that different treatment was performed, but that most of them used conservative treatment or percutaneous aspiration, resulting in poor pain release and high recurrence rate. Therefore, open surgery could provide complete cyst excision and sufficient decompression.^[17,18]^

Taking into account the patient’s age and physical condition, as well as imaging findings that show a primary manifestation of decreased intervertebral disc height and endplate inflammation, there is a risk of postoperative disc instability. Simple decompression through a single portal may necessitate a second surgery, which is undesirable for the patient. Therefore, we have abandoned the minimally invasive surgical approach and instead adopted the surgical technique of Posterior Lumbar Interbody Fusion with Endplate Decortication (PLED).

During the surgery, we found no cyst or any membranous structure around the right L5 nerve root, but we did find that left L5 spinal nerve root canal stenosis and herniated disc. According to the intraoperative findings and the medical history told by the patient, the epidural gas may be gas that accompanied the medicine injected when the patient underwent the previous “block surgery” for radicular pain. Since nitrogen is the main constituent of air and is hard to be absorbed by the human body, it could have remained trapped in situ.

Of course, the present paper reports only 1 case, and the issues of the pathogenesis, diagnosis, and management of pneumorachis remain unsolved. Nevertheless, this case highlights that in MRI examination, pneumorachis is similar to LDH and that pneumorachis may occur at the same level as LDH or lumbar stenosis, leading to misdiagnosis and complicating management decision making. In addition, there are still some limitations in this study: the follow-up period for this patient was relatively short, and long-term postoperative follow-up was not maintained to understand the more distant prognosis.

## 4. Conclusion

For patients with suspected spinal pneumatosis, CT scans hold significant value to distinguish air and bones from soft-tissue lesions. Furthermore, in this case, we have demonstrated the effectiveness of open surgical treatment for spinal pneumatosis and achieved favorable prognostic outcomes.

## Acknowledgments

This work was funded by Jilin Provincial Department of Science and Technology (YDZJ202301ZYTS101).

## Author contributions

**Investigation:** Yadong Liu.

**Resources:** Yadong Liu, Feng Xu.

**Writing – original draft:** Songning Fu.

**Writing – review & editing:** Songning Fu, Lu Liu, Feng Xu.
